# Deep Reinforcement Meta-Learning and Self-Organization in Complex Systems: Applications to Traffic Signal Control

**DOI:** 10.3390/e25070982

**Published:** 2023-06-27

**Authors:** Marcin Korecki

**Affiliations:** ETH Zurich, Computational Social Science, 8092 Zurich, Switzerland; marcin.korecki@gess.ethz.ch

**Keywords:** complex systems, self-organization, meta-learning

## Abstract

We studied the ability of deep reinforcement learning and self-organizing approaches to adapt to dynamic complex systems, using the applied example of traffic signal control in a simulated urban environment. We highlight the general limitations of deep learning for control in complex systems, even when employing state-of-the-art meta-learning methods, and contrast it with self-organization-based methods. Accordingly, we argue that complex systems are a good and challenging study environment for developing and improving meta-learning approaches. At the same time, we point to the importance of baselines to which meta-learning methods can be compared and present a self-organizing analytic traffic signal control that outperforms state-of-the-art meta-learning in some scenarios. We also show that meta-learning methods outperform classical learning methods in our simulated environment (around 1.5–2× improvement, in most scenarios). Our conclusions are that, in order to develop effective meta-learning methods that are able to adapt to a variety of conditions, it is necessary to test them in demanding, complex settings (such as, for example, urban traffic control) and compare them against established methods.

## 1. Introduction

As we learn more about the many systems that make up our world, the more complex it appears to become. The economy, biosphere, society, power grids, cities, and urban traffic are just a few examples of systems beyond our ability to fully understand, predict, or control.

A proposed response to the limitations of our understanding has recently come in the form of black box models such as deep learning. In complex systems, deep reinforcement learning (RL) methods have been especially favored and frequently used in control problems. There are many benefits to using deep learning, including its universality and ease with which it can be applied to a variety of problems. Indeed, in many tasks, deep learning has been shown to perform better than humans [1,2].

Nevertheless, there are also limitations to what deep learning methods can do. Some of the most commonly mentioned limitations include lack of robustness, adaptability, explainability, and fairness [3]. Most of the deep models do not currently contribute to a good understanding of the various systems they might help us control. Generally, we do not understand traffic any better by knowing that a particular deep learning model would control it in a certain way under some particular conditions.

In actuality, a deep learning model is an additional complex system we fail to fully understand. It might appear somehow unfounded to create a complex system, the inner workings of which we do not fully grasp, to control another complex system we do not entirely understand. This also makes it difficult to accurately assess the deep model’s success in an absolute sense, since we do not usually know the optimal behavior of the system we are attempting to control. To understand and interpret the performance and utility of deep learning models, it is useful to compare them against non-deep learning models that are well-studied and -understood.

Moreover, as dynamic complex systems continue to evolve in unpredictable ways, classical deep learning approaches often become inadequate as they cannot function effectively under significant data-shifts. Hence, the more adaptable meta-learning methods have become popular when applying deep learning to complex systems [4].

A real-world example of a complex system is urban traffic. Traffic congestion is an emergent property of most, if not all, sufficiently large modern metropolises. It is mostly considered as a negative emergent and leads to lost time, air and sound pollution, and frustration. Any truly smart city would hope to resolve the issue or at least mitigate it. Currently, the main way of dealing with congestion is through the design of smart traffic signal control approaches.

A great variety of RL multi-agent systems have been recently designed for control of traffic signals in urban environments [5,6,7], including ones that are able to meta-learn [8,9]. However, the majority of these methods have only been compared against previous RL methods (which they are usually shown to outperform). While this shows that deep learning for traffic signal control is continuously getting better, it is not yet clear how well it actually performs with regard to other approaches, such as analytic ones.

In this paper, we study the advantages and limitations of deep learning approaches for the problem of traffic signal control in the complex system of urban traffic. We conducted experiments on traffic signal control in simulated urban networks to compare RL methods with an analytic self-organization approach. We highlight that, while using deep learning in the context of complex systems has much potential, there is a need for strong baselines agains twhich to compare. Lastly, we conclude that, in the context of traffic signal control and at the current state of deep learning development, self-organization appears to be a good baseline, which is able to outperform RL methods in many settings.

## 2. Motivation

In this section, we discuss the relevant motivation, delineate key concepts, and present some background needed to grasp our experiments.

### 2.1. Deep Learning

Deep learning refers to any model that uses a Deep Neural Network (DNN). In most cases, the DNN is trained with some data and used as a classifier or decision maker. Deep learning does not require any specialist knowledge about the system for which it is used (but may still incorporate it in certain models [10,11]). Deep learning can be widely applied to a large variety of different systems; for example, the same DNN architecture can be used to learn to control traffic or a power grid.

On the other hand, the training of most deep learning models requires large, representative datasets and is computationally demanding [12]. The DNN is itself a complex structure, often containing thousands of parameters across many layers. It can itself be studied as a complex system [13] (where, from the local interactions of individual nodes, there emerge some global activation patterns). Due to its great number of unexplainable parameters, deep learning has recently been criticized for lacking fairness [14] and being prone to biases induced by training data [15]. Some of the limitations of deep learning have been linked to the strong influence on the field by the practice of engineering [3].

In traffic systems, deep RL has been applied to the problem of traffic signal control [16]. A variety of methods have been proposed, including [5,9].

### 2.2. Meta-Learning

In the context of learning conditions—where the underlying system might undergo an unpredictable change—or using data—in which distribution might shift—meta-learning has been seen as a promising methodology. Generally, meta-learning methods, unlike traditional deep learning, are able to be generalized to scenarios different from the one they were trained on [17]. In this context, a common method is to use gradient-based model agnostic meta-learning, periodically alternating between a global and individual adaptation [4]. One might also benefit from using mixed experience replays based on different scenarios [8]. Similarly, improved generalization can be gained from using model agnostic meta-learning and flow clustering [9]. Meta-learning can also be used to improve data efficiency, which then allows one to limit the interactions with the environment necessary for convergence [18]. Incorporating short- and long-term information by employing decentralized advantage actor–critic [19] can also lead to better generalization ability.

It is worth noting that, of the meta-learning methods proposed recently, many are highly complex. While showing great promise in dealing with the issues of adaptability to changing conditions, which are especially pertinent in complex systems, the meta-learning methods have mostly been compared to other learning methods [8,9]. It is the interest of this paper to also offer a comparison with non-learning methods that can be used as a simple baseline for future comparisons.

### 2.3. Complex Systems

A complex system is usually defined as a system composed of many sub-systems that interact with each other [20]. Such a system may be difficult to model, as the relationships between the parts are often non-linear and high-dimensional. For the same reason, such a system is also difficult to control. It may not even be clear how a given local intervention or action will affect the global state of the system. A concept often associated with complex systems is emergence, which is a spontaneous appearance of a global pattern precipitated by the local interactions of the system’s parts [21]. Since complex systems exhibit high levels of unpredictability and change, meta-learning is the best-suited learning paradigm for such settings.

Urban traffic can be seen as an instance of a complex system. It consists of many individual systems: intersections, vehicles, and pedestrians, all interacting with each other non-linearly [22].

### 2.4. Self-Organization

We follow a working definition of self-organization, wherein it is an adaptive process through which a system can acquire and maintain structure in the absence of external control [21]. Due to its decentralized character, such self-organized order exhibits high resilience to perturbations [23]. In social sciences, self-organization is also known as spontaneous order or invisible hand phenomenon, which characterizes liberal free-market economies [24]. Certain phenomena might also be considered to be self-organizing or not, depending on the perspective of the observer [25]. As such, self-organization is not a control approach, but rather a paradigm that aims to achieve desirable properties of the system, by creating favorable conditions for their emergence. Many definitions of self-organization have been given in the literature (see [21] for an overview); however, in this work, we do not follow a strict definition (only a working one, given above), but, rather, consider self-organization in the particular context of a complex system, where local interactions lead to a certain order being established at a global level. In Table 1, we present a comparison between deep learning and self-organization approaches in the context of complex systems.

An emergent self-organizing property observable in urban traffic would be the synchronization of neighboring signals, resulting in a ‘green wave’. An example of unfavorable self-organization, in contrast, would be the formation of traffic jams. A self-organizing traffic control aiming to overcome such congestion would be based on a set of modified interaction rules that are followed by individual agents in a decentralized manner (as, for example, in [26]).

### 2.5. Problem Description

In this work, we compare the effectiveness of deep learning and self-organization for controlling the traffic signals in an urban traffic system. The traffic system presents a challenging problem for deep learning algorithms as the traffic conditions can be highly dynamic and difficult to predict. A model which is trained only on morning traffic will likely under-perform on evening traffic. On the other hand, training a model to expect certain temporal patterns (e.g., morning vs. evening) might produce a model, which will not work well when an unexpected accident or roadwork causes the traffic patterns to change unexpectedly. Due to its high sensitivity, dynamic nature, and low predictability, the urban traffic system and its control are a difficult but interesting problem for the domain of deep learning. As we have mentioned, the particular approach that appears highly relevant to such a setting is meta-learning, which offers to produce models that are able to adapt and work well, even in changing conditions.

**Figure 1:**
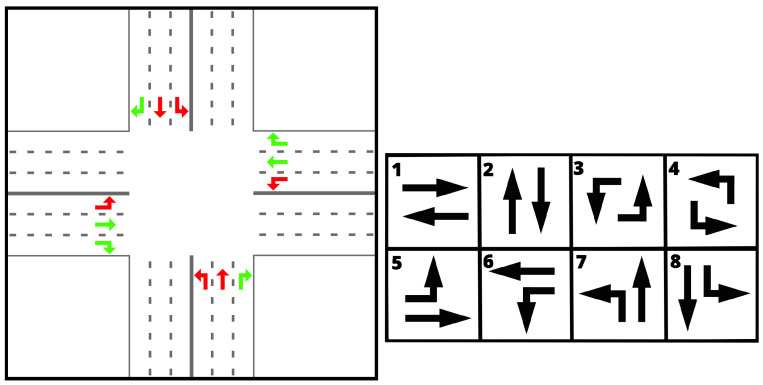
**Left**: An intersection with eight flows (not counting the right turns, which are assumed to always be given green in any phase). The green arrows correspond to the flows that would be given green by phase 1 from the right figure, red arrows represent flows that would be given red. **Right**: The possible actions (phases) available to an intersection agent controlling an intersection with eight flows (indicated by number 1 through 8).

Generally, the traffic signal control problem consists of individual intersections (agents) taking *actions*. The *actions* consist of *flows* (traffic moving through an intersection from one lane to another) aggregated into *phases* (a phase represents the set of all flows that are given green, see Figure 1). The goal is to optimize a certain metric (often, the average travel time) to avoid or delay congestion.

The learning agents typically used to control signals at an intersection are based on deep reinforcement learning algorithms. The design of these agents can be defined by the reinforcement learning paradigm they use (e.g., *q*-learning or policy gradient), the actions, states, and reward. The actions available to such an agent correspond to phases that are possible at the intersection. An example of possible actions for an intersection with eight flows is given in Figure 1. The states can vary in detail (depending on the particular implementation) but correspond to the meaningful features of the traffic situation at the intersection. As such, they usually contain information such as the number of vehicles on the lanes (occupancy), the current phase, and so on. The reward corresponds to the measure of success and usually involves average travel time, delays, or number of stops. A description of the design of the intersection agents used in our experiments can be found in Section 3.

## 3. Methods

In this section, we introduce the main methods of the study—the Analytic+—and describe the details of the simulation experiments, including the reinforcement learning methods we compare against.

### 3.1. Analytic+

The Analytic+ algorithm [27] is based on short term anticipation of traffic. It relies on two rules: an optimization rule and a stabilization rule. The optimization rule is designed to minimize the total waiting times at a given intersection, while the stabilization rule maintains the stability and fairness of the service by ensuring that no flow incurs waiting times above a certain maximum. The Analytic+ method is decentralized, meaning the agents do not communicate with each other and make decisions based only on locally available information. This local information does, however, depend on the decisions taken by neighboring intersections; thus, an implicit communication between neighbors does occur. Each agent follows only two explainable rules. Despite the simplicity, from the actions of each agent emerges a global, self-organizing order. The algorithm has carefully designed explainable parameters that define the average and maximum waiting times allowed by the stabilization rule. The pseudocode for the algorithm is presented in Algorithm 1, and the code of the implementation used for this study is available at https://github.com/mbkorecki/meta_traffic (accessed on 26 June 2023).
**Algorithm 1:** Analytic+ pseudocode.
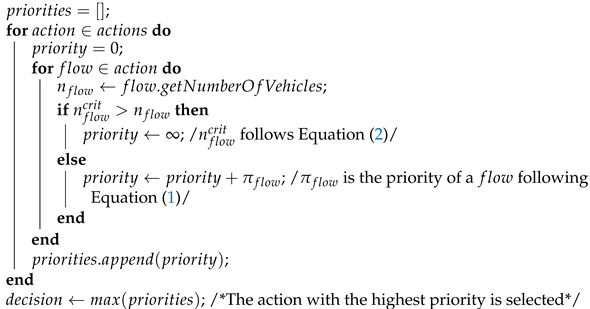


#### 3.1.1. Optimization Rule

The optimization rule prioritizes the flows with highest priority scores according to Equation (1):

(1) $$\pi_{i}=\frac{{\hat{n}}_{i}}{\tau_{i,\sigma }^{pen}+\tau_{i}+{\hat{g}}_{i}}.$$

Herein, ${\hat{g}}_{i}$ is the required green time needed to clear lane *i* (this is a variable dependent on the number of cars on *i* and their maximum legal speed), $\tau_{i}$ is the penalty for switching to another traffic flow, $\tau_{i,\sigma }^{pen}$ is the penalty for switching back, and ${\hat{n}}_{i}$ is the number of vehicles that are expected to be served in time period ${\hat{g}}_{i}+\tau_{i}$. This formulation ensures that the total waiting times of the vehicles at an intersection are minimized (assuming an optimization horizon that includes only these vehicles, whose waiting time depends on the current traffic signal).

#### 3.1.2. Stabilization Rule

The stabilization rule is based on two parameters $T_{avg}$ and $T_{max}$, indicating the average waiting time and the maximum waiting time that can be incurred by each flow. These parameters can be specified separately for each intersection or homogeneously throughout the entire system. Using $T_{avg}$ and $T_{max}$, we define a critical threshold $n_{i}^{crit}$. When the number of vehicles waiting for a given flow surpasses $n_{i}^{crit}$, the flow is added to a priority queue and is served in the next service period. The $n_{i}^{crit}$ is fully specified in Equation (2), where $Q_{i}$ indicates the average arrival rate of flow *i*, and $z_{i}$ is the variable service interval between two successive service processes for *i*:

(2) $$n_{i}^{crit}=Q_{i}T_{avg}\frac{T_{max}-z_{i}}{T_{max}-T_{avg}}.$$

### 3.2. Simulation Experiments

Two main components of the experiments were the analysis of the analytic approach—the effects of its paramterization on performance—and its comparison with state-of-the-art RL methods. The experiments were conducted in Cityflow [28]. The Cityflow simulator was selected for its high-speed performance (as compared to, for example, SUMO). In addition, most of the recent deep learning methods applied to traffic signal control (including the methods we reported in our experiments) have been implemented and tested in this particular simulator.

For the comparison experiments, we were mostly interested in the adaptability of the compared methods. The adaptability was expressed in terms of consistency of performance across a variety of scenarios (increasingly different from the training scenario, in the case of learning methods). Furthermore, the consistency of the performance across a given flow distribution was also of interest. Hence, we report the variation in performance in terms of the maximum and minimum values achieved on a given flow distribution by a given method.

### 3.3. Scenarios

**Figure 2:**
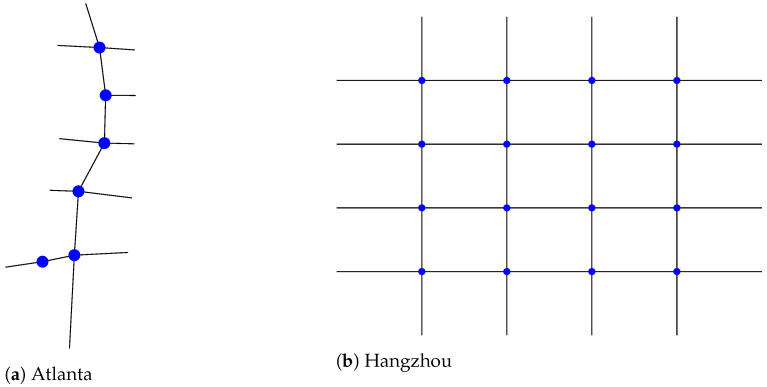
The road networks of the two studied scenarios. The black lines represent roads, the blue dots intersections.

We ran the experiments on two scenarios representing parts of Atlanta and Hangzhou; the road networks are visualized in Figure 2. Note that Atlanta had a much more irregular network, while Hangzhou had a regular grid. Moreover, intersections in the Atlanta scenario were heterogeneous (connecting four, three, and two roads), while, in the Hangzhou road network, all intersections were the same (connecting four roads). We chose these scenarios because they allowed us to test the methods in both heterogeneous and homogeneous settings. Furthermore, they have been used in many previous publications on these topics, making it easier to compare across a spectrum of publications and methods [5,8,9].

In real world traffic systems, the flows change at different time scales (minutes, hours, days, months). They might also be affected by unpredictable events such as accidents or road work. Clearly, one of the key characteristics of a good traffic signal control method is how well it can adapt to changing traffic conditions. Specifically, RL methods must be tested in conditions that are different from the ones they were trained on. Therefore, to test the adaptability of the compared methods, we generated a variety of traffic flows for each of the two scenarios. These flows can be understood as representing different traffic conditions that may result, for instance, due to disruptions or different times of day.

We followed the methodology from [9], where a Wasserstein generative adversarial network (WGAN) was used to generate traffic flows from distributions away from the training distribution (which is based on real world data). To make sure that the generated traffic flows were realistic enough, some constraints were enforced through the loss function. For example, the variation in the count of vehicles between two adjacent time intervals could not differ too much. The exact details of the implementation can be found in [9].

We generated four traffic flow distributions away from the training distribution by 0.005, 0.01, 0.05, and 0.1, in terms of Wasserstein distance. For each distribution, we generated ten flows, tested on all of them, and reported minimum, maximum, and mean average travel times achieved by each method. In Table 2, we present the details of the scenarios. As the Wasserstein distance from the initial training distribution increased, the median entry time into the system decreased, leading to more vehicles being present in the system at the same time, which increased the complexity of the system and difficulty of controlling it. The number of vehicles also increased with the Wasserstein distance, which again led to more complex traffic dynamics. The increase in the number of vehicles was more pronounced for the Hangzhou scenario.

To study the effects of parameter settings on Analytic+, we ran the method on all scenarios with five different parameter settings and reported the mean average travel times achieved for each of the flow scenarios (ten different flows from each distribution). The parameter settings were selected according to their meaning. The first parameter indicated the average wait time, while the second indicated the maximum wait time. Therefore, they needed to be sufficiently different (with the second being higher) in order to make sense. Generally, the lower the maximum waiting time, the better for the individual drivers (but not necessarily better for the system’s global performance). We searched through a varied range of parameters, from low (better for individuals) to high (potentially better for the system). The results for the RL methods were taken from [9].

### 3.4. Compared Methods

In our studies, we compared the results of the Analytic+ against eight RL methods. The full results are available in Appendix A, but, for the sake of brevity, we only present the results of the three best performing methods in Section 4. These methods were chosen because they were reported to have achieved state-of-the-art results when published and were compared with many other learning algorithms (but not analytic baselines, which was important for this paper). Moreover, the Generalight and Metalight methods explicitly used a form of meta-learning, which was of particular interest and was highly applicable to our problem statement. The hyperparameters of the learning methods and their implementations have been given in https://github.com/only-changer/GeneraLight, this repository (accessed on 26 June 2023), which follows [9]. The best hyperparameters for each algorithm were assumed to have been worked out in [9] or in each publication introducing the corresponding method, as referenced below. We described each agent in terms of its states, rewards, and additional details. The actions of each agent were the same and correspond to the phases given in Figure 1.

Specifically, the algorithms are listed below:

- **Analytic+**: The self-organizing method described in Section 3.1;
- **Generalight**: A meta-RL method based on a deep *q*-learning paradigm, using the MAML framework [4] and clustering [9]. It outperforms other RL methods in scenarios that are different from the training scenarios. States: one-hot encoded vector representing the current phase at the intersection and the numbers of vehicles on each incoming lane and outgoing lane. Reward: the negative of the pressure of the intersection, which is a metric measuring the imbalance between outgoing and incoming lanes [7];
- **Colight**: An RL method based on a deep *q*-learning paradigm, designed to handle multi-intersection environments and using attention mechanism to share information between neighbors. States: one-hot encoded vector representing the current phase at the intersection and the number of vehicles on each incoming lane. Reward: the sum of queue lengths of each incoming lane [5];
- **Metalight**: This method transfers knowledge between intersections, designed to generalize to intersections with varied numbers of incoming and outgoing lanes. States: one-hot encoded vector representing the current phase at the intersection and the number of vehicles on each incoming lane. Reward: the average queue length of incoming lanes [8].

It is worth noting that the **Analytic+** method can be easily scaled to as large a number of intersection as is needed. Since it does not require training, the cost of deployment is constant. The cost of operation is low since the algorithm does not perform any costly computations. On the other hand, the deployment cost of the learning algorithms is higher as they need to be trained for each specific scenario.

## 4. Results

**Figure 3:**
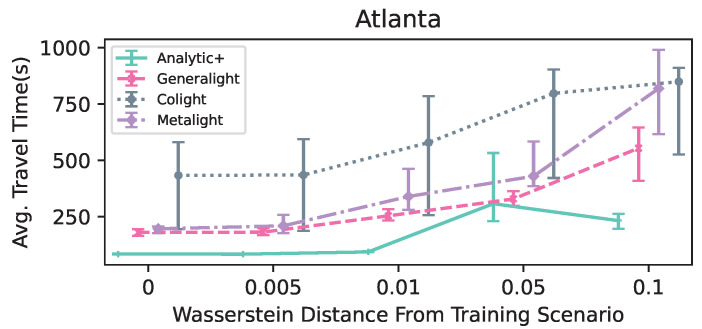
The performance, in terms of average travel time (seconds), of the compared methods in the Atlanta scenarios. The variability intervals represent the minimum and maximum values achieved for a given set of scenarios; the mean is marked on the line. Lines are guides to the eye only, to indicate trends.

**Figure 4:**
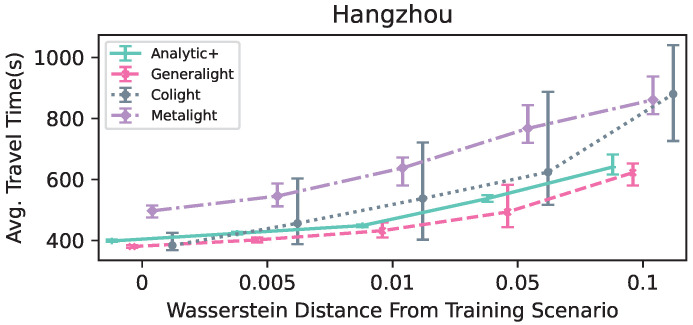
The performance, in terms of average travel time (seconds), of the compared methods in the Hangzhou scenarios. The variability intervals represent the minimum and maximum values achieved for a given set of scenarios; the mean is marked on the line. Lines are guides to the eye only, to indicate trends.

In this section, we report the results of the parameter study (Table 3 and Table 4), as well as the performance of the studied methods in the two scenarios (Figure 3 and Figure 4).

### 4.1. Analytic+ Parameter Study

Table 3 shows that, for the Atlanta scenarios, a good choice of parameters (w.r.t. avg. travel time) was $T_{avg}=120$, $T_{max}=240$. This setting produced the lowest avg. travel times in three out of five flow scenarios. Note that the differences for flows $D_{0}$, $D_{0.005}$, and $D_{0.01}$ were minimal; they increased for $D_{0.05}$ and were even greater for $D_{0.1}$. $T_{avg}=60$, $T_{max}=120$ was by far worse than the other settings, with $D_{0.01},$ $D_{0.05}$, and $D_{0.1}$.

Based on Table 4, the optimal parameters for the first three flow scenarios of the Hangzhou scenarios were T=∞, $T_{max}=\infty$, and, for the last two, T=240, $T_{max}=360$. The difference between the two parameter settings for the first three flow scenarios was very small (around 1%). For the last two flow scenarios, they were larger (around 3%). Since we considered the last two scenarios to be more complex (because they were most different from the training scenario), we preferred to use T=240, $T_{max}=360$ as the optimal parameter settings.

Note that the optimal parameter setting for the Hangzhou scenarios is different from the Atlanta scenarios, showcasing that these parameters are dependent on the flows and road networks. In the following, we used T=120, $T_{max}=240$ for Atlanta and T=240, $T_{max}=360$ for Hangzhou.

### 4.2. Comparison Study

Figure 3 shows the average travel time achieved in each of the compared methods on the Atlanta scenarios. In all five flow scenarios, the Analytic+ achieved better mean performance, in most cases with relatively tight variability intervals. It allows for approximately two times lower travel times in four out of five flow scenarios when compared to Generalight—the next-best method. Generalight and Metalight achieved similar results for the first three scenarios but diverged for the last two scenarios, where Generalight was superior. Moreover, Analytic+ also had the smallest variability intervals in four out of five flow scenarios, meaning it performed more consistently over scenarios sampled from the same distribution than the other methods.

Figure 4 displays the performance of the compared methods for the Hangzhou scenarios in terms of average travel time. Here, Analytic+ performed at the same level as Generalight in all five flow scenarios. Colight was significantly worse than both Generalight and Analytic+. Again, Analytic+ displayed the smallest variability intervals for all flows. Metalight performed worse than the other two RL methods, unlike in the Atlanta scenario, where it performed better than Colight.

Exact numerical results, along with comparison with additional methods, are available in Appendix A.

## 5. Discussion

The immediate impression from our results is that reinforcement learning methods do not appear to handle heterogeneity well. While Analytic+ and Generalight performed at a comparable level in the homogeneous, grid-like Hangzhou street network, Analytic+ was twice as good as Generalight in the heterogeneous artery of Atlanta. This might occur due to different lengths of incoming lanes in Atlanta scenario, as has been observed in [29]. Moreover, the heterogeneity of intersection (difference in the number of incoming lanes) might negatively affect RL’s ability to generalize [8]. Analytic+ does not appear to be affected—it can deal well with heterogeneity, both in terms of lane lengths and the number of lanes.

Moreover, in both scenarios, Analytic+ had the smallest variation in performance, indicating that it performs consistently well over any flow drawn from a given distribution. The same was not true for RL agents, some of which were strongly inconsistent, even across flows drawn from the same distribution (e.g., Colight). It is also clear (Appendix A) that most of the RL methods, other than Generalight, performed at a sub-optimal level in both scenarios across all distributions.

One of the limitations of this study is that it only compares the performance in terms of one metric—the average travel time. Nevertheless, there are other important metrics that can be taken into account, including waiting times (which can be optimized by the Analytic+ stabilization rule; (as such, Analytic+ is expected to perform very well again). A multi-objective approach to traffic optimization and comparison of methods across more than one metric will be a future direction of research.

Furthermore, a potential future extension is to learn the optimal parameters of the Analytic+ using RL methods. Thus, we could combine the benefits of deep learning with the benefits of the self-organization. Since Analytic+ parameters are explainable, the results learned by an RL agent could be easily validated.

Nevertheless, this traffic signal control problem seems highly relevant to the complexity as well as machine learning community. It provides a real-world example of a complex system which proves highly challenging for traditional deep learning as well as meta-learning algorithms. The need for lifelong adaptation is also clear in the traffic system, where unpredictable events such as accidents, jams, and roadwork can occur daily (the effects of such disruptions on learning algorithms in the context of traffic has been investigated in [30]). In order for a traffic signal control algorithm to be actually useful, the learning algorithm would need to enable continuous learning and be highly adaptable.

Moreover, this study raises a question of the applicability of deep learning to control of complex systems in general. This question should certainly be further studied; the most promising methodologoies appear to be meta-learning approaches. The non-learning baseline that we provide makes it easier to propose and compare new effective algorithms for this particular problem setting.

In conclusion, we have investigated the applicability of deep learning and self-organization methods to complex systems, exemplified by an urban traffic system. We have shown that the self-organization method can achieve better or comparable results to deep learning, while not requiring any training, in addition to being explainable and fair. Apparently, there is more work to be performed if deep learning is to be used in the field of complex systems. Clearly, deep learning has great potential (especially meta-learning, in the context of complex systems), but, in order for it to be fully realized, it needs to be approached critically and challenged against strong, non-deep learning baselines. We have provided an approachable setting in which the performance of learning and non-learning control methods can be studied in a complex system. We have also provided a strong, non-learning baseline for this system. We hope that our contribution will make it more accessible for future researchers to study and design learning approaches that can work well in the challenging domain of complex dynamic systems.

## Figures and Tables

**Table 1:** Differences between deep learning and self-organization applied to complex systems.

Deep Learning	Self-Organization
Requires **training** on large and representative datasets	Ideally **no training** and no or little data requirements
Might need **retraining** to adapt if the system evolves in a way such that the training data are not representative	Can adapt to an evolution that the system might undergo and needs **no retraining**
Needs an extremely large set of parameters, which are **not explainable**	Typically uses a limited number of **explainable** parameters
Might be **lacking fairness**	**Fairness** can be promoted/guaranteed
**Requires no specialist knowledge** or limited knowledge of the system	Might **require specialist knowledge** about the system to design effective rules
Can be **universally applied** to a variety of different systems	**Application is limited** to specific kinds of systems which are able to self-organize

**Table 2:** The number of vehicles and their median entry time (s) into the system for all studied scenarios. Median entry time represents the time at which half of all the vehicles have entered the system.

	Number of Vehicles					Median Entry Time (s)				
**Scenario**	$D_{0}$	$D_{0.005}$	$D_{0.01}$	$D_{0.05}$	$D_{0.1}$	$D_{0}$	$D_{0.005}$	$D_{0.01}$	$D_{0.05}$	$D_{0.1}$
Atlanta	2530	2570	3060	3150	3120	1928	1938	1856	1846	1676
Hangzhou	5397	6447	7434	7875	8106	1941	1923	1894	1838	1680

**Table 3:** Mean of the average travel times in seconds achieved by the Analytic+ algorithm in the Atlanta scenarios for different parameter settings. $D_{.}$ indicates the traffic flow type of the scenario in terms of the Wasserstein distance from the $D_{0}$ scenario. The best (lowest) average travel times are shown in bold. Parameters set to *∞* correspond to the stabilization rule not being applied.

	Avg. Travel Time(s)				
Parameter Settings	$D_{0}$	$D_{0.005}$	$D_{0.01}$	$D_{0.05}$	$D_{0.1}$
$T_{avg}=60$, $T_{max}=120$	85.5	86.0	393.1	664.2	1113.5
$T_{avg}=120$, $T_{max}=240$	84.0	**83.9**	**93.8**	307.8	**232.6**
$T_{avg}=240$, $T_{max}=360$	**83.8**	84.2	93.9	289.4	315.1
$T_{avg}=360$, $T_{max}=480$	83.9	84.0	93.9	**276.1**	353.0
$T_{avg}=\infty$, $T_{max}=\infty$	83.9	84.0	93.9	292.3	363.7

**Table 4:** Mean of the average travel time in seconds achieved by the Analytic+ algorithm in the Hangzhou scenarios for different parameter settings. $D_{.}$ indicates the traffic flow type of the scenario in terms of the Wasserstein distance from the $D_{0}$ scenario. The best (lowest) average travel times are shown in bold. Parameters set to *∞* correspond to the stabilization rule not being applied.

	Avg. Travel Time(s)				
Parameter Settings	$D_{0}$	$D_{0.005}$	$D_{0.01}$	$D_{0.05}$	$D_{0.1}$
$T_{avg}=60$, $T_{max}=120$	542.2	645.5	763.1	851.1	995.8
$T_{avg}=120$, $T_{max}=240$	438.0	504.4	625.9	735.4	842.5
$T_{avg}=240$, $T_{max}=360$	398.4	423.6	448.8	**537.5**	**640.9**
$T_{avg}=360$, $T_{max}=480$	400.8	425.3	445.0	554.1	648.9
$T_{avg}=\infty$, $T_{max}=\infty$	**396.7**	**417.0**	**442.8**	557.3	660.8

## Data Availability

Not applicable.

## Appendix A. Additional Results

Here, we present the full results of the comparison experiments. The additional methods are listed here:

- **DQN**: Simple deep *q*-network approach. Uses vehicles’ positions as states [6];
- **PressLight**: State-of-the-art RL method. Uses number of vehicles on incoming and outgoing lanes, current phase as state, and pressure as the reward [7];
- **+MAML**: Adds the MAML RL framework to a given method [4].

**Table A1:** Performance of all methods in the Atlanta scenarios, in terms of average travel time (seconds). The best methods are shown in bold.

Model	Avg. Travel Time(s)														
		$D_{0}$			$D_{0.005}$			$D_{0.01}$			$D_{0.05}$			$D_{0.1}$	
	**Max**	**Min**	**Mean**	**Max**	**Min**	**Mean**	**Max**	**Min**	**Mean**	**Max**	**Min**	**Mean**	**Max**	**Min**	**Mean**
DQN	301.7	255.5	280.3	297.1	241.8	266.1	577.1	349.1	399.9	690.1	375.5	441.9	991.3	578.3	755.3
+MAML	264.3	201.3	226.5	237.8	194.0	214.8	517.9	299.2	344.8	641.9	345.0	414.5	983.2	525.3	742.1
PressLight	472.0	207.2	286.9	477.3	198.0	277.6	561.0	297.0	346.2	631.6	383.3	430.4	968.0	684.4	859.7
+MAML	223.0	189.5	210.1	230.9	195.8	218.5	370.8	266.2	306.4	**435.7**	361.9	394.3	994.5	551.8	669.8
MetaLight	206.9	176.8	196.5	258.4	177.0	209.6	463.1	279.9	338.9	583.3	386.3	429.9	991.2	616.5	819.2
CoLight	580.7	195.5	433.3	593.6	187.1	435.2	785.4	256.6	579.1	903.4	421.9	797.5	911.1	526.5	849.2
+MAML	632.6	140.9	427.3	600.0	190.2	290.4	869.4	255.1	558.7	919.4	580.5	794.0	992.9	527.1	847.3
GeneraLight	**194.5**	**165.1**	**180.3**	**200.0**	**167.7**	**181.6**	**283.4**	**232.8**	**253.9**	**363.3**	**298.5**	**329.0**	**646.6**	**409.0**	**554.2**
Analytic+	**86.5**	**82.9**	**84.0**	**85.8**	**82.0**	**83.9**	**96.7**	**92.5**	**93.8**	532.8	**230.3**	**307.8**	**263.3**	**196.7**	**232.6**

**Table A2:** Performance of all methods in the Hangzhou scenarios, in terms of average travel time (seconds). The best methods are shown in bold.

Model	Avg. Travel Time(s)														
		$D_{0}$			$D_{0.005}$			$D_{0.01}$			$D_{0.05}$			$D_{0.1}$	
	**Max**	**Min**	**Mean**	**Max**	**Min**	**Mean**	**Max**	**Min**	**Mean**	**Max**	**Min**	**Mean**	**Max**	**Min**	**Mean**
DQN	979.1	846.6	921.0	1016	935.7	986.7	1069	964.1	1010	1127	1024	1088	1284	1209	1249
+MAML	530.1	394.8	448.2	605.0	462.0	529.5	707.9	504.2	617.0	855.8	715.7	781.0	1147	884.4	1021
PressLight	395.9	384.6	389.9	435.8	**415.7**	426.6	484.2	436.2	460.3	662.0	565.7	592.9	855.1	749.2	813.4
+MAML	468.1	395.6	424.0	597.9	468.9	520.5	712.4	569.1	634.5	909.8	665.9	801.4	1100	817.0	1004
MetaLight	514.4	475.0	497.2	587.3	512.0	545.6	671.6	580.5	637.8	843.3	719.9	767.4	937.9	814.2	861.1
CoLight	425.6	**368.7**	**383.9**	603.0	387.7	456.4	721.5	**402.7**	537.8	887.9	517.1	624.1	1040.4	726.2	880.2
+MAML	**402.7**	390.9	397.5	490.4	424.8	452.0	531.7	458.6	482.9	643.1	**513.0**	570.2	785.4	638.0	718.3
GeneralLight	**385.1**	**376.3**	**380.0**	**410.2**	**393.6**	**402.0**	**458.7**	**409.4**	**432.2**	**582.0**	**443.5**	**493.5**	**652.2**	**579.8**	**622.5**
Analytic+	403.2	395.4	398.4	**428.3**	419.3	**423.6**	**452.0**	444.2	**448.8**	**548.5**	526.4	**537.5**	**682.3**	**616.3**	**640.9**
